# Longitudinal association of remnant cholesterol with joint arteriosclerosis and atherosclerosis progression beyond LDL cholesterol

**DOI:** 10.1186/s12916-023-02733-w

**Published:** 2023-02-06

**Authors:** Zhiyuan Wu, Jinqi Wang, Haiping Zhang, Huiying Pan, Zhiwei Li, Yue Liu, Xinlei Miao, Ze Han, Xiaoping Kang, Xia Li, Xiuhua Guo, Lixin Tao, Wei Wang

**Affiliations:** 1 Beijing Municipal Key Laboratory of Clinical Epidemiology, Department of Epidemiology and Health Statistics, School of Public Health, Capital Medical University, No.10 Xitoutiao, Youanmen Street, Beijing, 100069 China; 2 Centre for Precision Health, Edith Cowan University, 270 Joondalup Drive, Joondalup, Perth, WA 6027 Australia; 3 Beijing Xiaotangshan Hospital, Beijing, China; 4 Department of Mathematics and Statistics, La Trobe University, Melbourne, Australia

**Keywords:** Remnant cholesterol, Arteriosclerosis and atherosclerosis, Brachial-ankle pulse wave velocity, Ankle brachial index, LDL cholesterol

## Abstract

**Background:**

Arteriosclerosis and atherosclerosis are closely related with cardiovascular disease (CVD) risk. Remnant cholesterol (RC) could predict CVD. However, its effect on joint arteriosclerosis and atherosclerosis progression remains unclear. This study aims to evaluate the association of RC with joint arteriosclerosis and atherosclerosis progression trajectories in the general population.

**Methods:**

This study collected data across five biennial surveys of the Beijing Health Management Cohort from 2010 to 2019. Multi-trajectory model was used to determine the joint arteriosclerosis and atherosclerosis progression patterns by brachial-ankle pulse wave velocity (baPWV) and ankle brachial index (ABI). We also performed discordance analyses for RC vs. low density lipoprotein cholesterol (LDL-C) using ordinal logistics model.

**Results:**

A total of 3186 participants were included, with three clusters following distinct arteriosclerosis and atherosclerosis progression patterns identified using a multi-trajectory model. In the multivariable-adjusted ordinal logistics analyses, RC was significantly associated with baPWV and ABI progression (OR: 1.20; 95% CI: 1.13–1.28, per 10 mg/dL). For the discordance analyses, the discordant low RC group was associated with decreased risk compared to the concordant group (OR: 0.73; 95% CI: 0.60–0.89). People with a high RC level were at an increased risk of joint arteriosclerosis and atherosclerosis progression, even with optimal LDL-C.

**Conclusions:**

RC is independently associated with joint arteriosclerosis and atherosclerosis progression beyond LDL-C. RC could be an earlier risk factor than LDL-C of arteriosclerosis and atherosclerosis in the general population.

**Supplementary Information:**

The online version contains supplementary material available at 10.1186/s12916-023-02733-w.

## Background

Cardiovascular disease (CVD) has become one of the greatest threats to public health and the leading cause of mortality and health burden worldwide [1]. Systemic arteriosclerosis and atherosclerosis are robust predictors of CVD and overall death [2, 3], causing vascular damage by degenerating arterial elasticity and increasing pulse pressure [4]. Therefore, it is of significance to identify the primary risk factors and potential target(s) for the early recognition of arteriosclerosis and atherosclerosis. Brachial-ankle pulse wave velocity (baPWV) and ankle-brachial index (ABI) are two sensitive and non-invasive alternative indicators for arteriosclerosis and peripheral atherosclerosis widely used in population studies [5–7]. High baPWV and low ABI are independent predictors of cardiovascular events and mortality [6, 8]. Thus, combining the two indicators, baPWV and ABI, can comprehensively assess the CVD risk.

Abnormal lipid metabolism plays a key role in arteriosclerosis and atherosclerosis [9–12]. Lowering low-density lipoprotein cholesterol (LDL-C) primarily through statin is the leading therapy target for primary and secondary prevention of arteriosclerosis and atherosclerosis-related diseases [13, 14]. However, patients with a substantial reduction in LDL-C still have a considerable residual CVD risk [15, 16]. Atherogenic dyslipidemia, characterized by high levels of triglycerides and low concentrations of high-density lipoprotein cholesterol (HDL-C) with normal concentrations of LDL-C, is a common lipid disorder and one of the main causes of lipid-dependent residual CVD risk [17]. In view of the fact that several clinical trials found that HDL-C raising therapies did not significantly reduce the risk of CVD [18], recent research attentions have shifted to triglyceride-rich lipoproteins (TRLs). Human cells can generally degrade triglycerides but not cholesterol. Thus, we hypothesized that the cholesterol component carried on TRLs may be the main culprits for arteriosclerosis and atherosclerosis [17]. Remnant cholesterol (RC) represents the cholesterol content of TRLs, i.e., the intermediate density lipoprotein (IDL) and very low density lipoprotein (VLDL) in the fasting state. It also includes the extra chylomicron remnants in the non-fasting state [19]. There are studies showing that TRLs could penetrate through and accumulate on the arterial wall and subsequently cause foam cell formation, atherosclerosis, and low-grade inflammation [19–21]. Emerging evidence suggested that remnant cholesterol (RC) could contribute to CVD residual risk to a large extent [15, 19]. Both epidemiological and genetic studies have reported the causal association of RC with CVD and mortality [17, 20, 22–28]. Considering the progression of arteriosclerosis and atherosclerosis is an important early vascular feature in the occurrence of CVD, evidence about the association of RC with arteriosclerosis and/or atherosclerosis is still limited. Cross-sectional studies found that increased RC is significantly associated with baPWV alone [7, 29]. However, the longitudinal association of RC with the joint arteriosclerosis and atherosclerosis progression remains unclear.

Therefore, we aimed to jointly characterize the progression trajectory combining multiple examinations of baPWV and ABI during a 10-year follow-up and to evaluate the effect of RC on the arteriosclerosis and atherosclerosis progression in the general population. This study for the first time provided data about RC with joint baPWV and ABI progression for the early recognition of arteriosclerosis and atherosclerosis from a longitudinal cohort perspective.

## Methods

### Settings

For the current study, we used individual-level data from the Beijing Health Management Cohort (BHMC). The BHMC study was conducted based on physical examination populations from Beijing Xiaotangshan Examination Center and Beijing Physical Examination Center, which are two biggest health examination centers in Beijing, China. The participants were required to undertake regular physical examinations (height, weight, heart rate, blood pressure, ultrasound, arterial stiffness), face-to-face questionnaire survey, and blood sample collection under a uniform examination package. This cohort collected longitudinal key variables such as lipid profiles, chronic diseases status, and arterial stiffness measurements that are valuable for CVD prevention, treatment, and management. The study was approved by the Ethics Committee of Capital Medical University (grant number: 2020SY031) and Edith Cowan University (grant number: 2021-03164-WU). All participants provided written informed consent before taking part in the study.

### Study design and population

We examined the association between the baseline level of RC, other lipid parameters, and joint arteriosclerosis and atherosclerosis progression, with the baseline defined as visit 1 between 2010 and 2011. The health examination during 2012 to 2013 was then defined as visit 2, 2014 to 2015 as visit 3, 2016 to 2017 as visit 4, and 2018 to 2019 as visit 5. The measurements of baPWV and ABI data were extracted from all visits. All participants aged 25 years and above without a history of CVD at baseline were initially screened for inclusion. Individual-level records with any missing data of lipid profiles or baPWV or ABI measurements were excluded. Finally, a total of 3186 individuals with three or more subsequent visits were enrolled for the final analyses.

### Laboratory measurements

Fasting blood samples were stored and measured in the central laboratories of Beijing *Xiaotangshan* Examination Center and Beijing Physical Examination Center. Fasting glucose, serum total cholesterol, triglyceride, HDL-C, and LDL-C were directly measured using the Olympus Automatic Biochemical Analyzer (Hitachi 747; Tokyo, Japan). The coefficients of variation (CVs) were < 4.0% for triglyceride and HDL-C, < 3.0% for total cholesterol, and LDL-C and < 5.0% for fasting glucose. Non-HDL-C was calculated as total cholesterol concentration minus HDL-C. RC was defined as non-HDL-C minus the calculated LDL-C by the Martin equation [30]. The Martin-Hopkins method matches 1/180 factor by individual triglycerides and non-HDL-C levels to estimate the LDL-C level. Thus, RC could be calculated by non-HDL-C minus the estimated LDL-C level (Stata code: https://www.ldlcalculator.com/). The Martin-Hopkins method is recommended by the American Heart Association (AHA) guidelines and has been applied in many population studies. RC was alternatively estimated as non-HDL-C minus the calculated LDL-C by the Friedewald equation for triglycerides < 400 mg/dL (or non-HDL-C minus the directly measured LDL-C for triglycerides ≥ 400 mg/dL) in the sensitivity analysis. In this current study, Martin equation was used in the main analysis and Friedewald equation was adopted in the sensitivity analysis. High-sensitivity C-reactive protein (hsCRP) was also measured, as hsCRP is closely related with RC and contributes to the CVD risk [14].

### Discordance definition

We used two approaches to define discordance between LDL-C and RC. First, we defined discordance by the percentile distance between RC and LDL-C. The population was classified into three groups: discordantly low RC (RC percentile < LDL-C percentile by 10 percentile units), concordant RC and LDL-C (RC percentile minus LDL-C percentile within ± 10 percentile units), and discordantly high RC (RC percentile > LDL-C percentile by 10 percentile units) following the previous study [26]. Second, we used the relevant clinical cut-off points to define discordance between LDL-C (100 and 130 mg/dL) and RC (17 and 24 mg/dL) according to the established Guideline Recommendations [31]. Third, we used the median values of LDL-C and RC as cut-off points [32, 33].

### BaPWV and ABI measurements

The baPWV was measured with an Omron Colin BP-203RPE III device (Omron Health Care, Kyoto, Japan). After a minimum of 5 min rest in the supine position, four cuffs were wrapped around the bilateral brachial and ankles and then connected to a plethysmographic sensor and oscillometric pressure sensor. The final baPWV was calculated as the length between the brachium and ankle divided by the transit time between the wave front of the brachial waveform and the ankle waveform [34]. ABI was calculated using the following formula [35]: ABI = SBP of posterior tibial artery/SBP of the brachial artery. At least two acceptable measurements were performed on each side (right and left) and the difference between two measures of each side should be less than 50 cm/s for baPWV and 0.05 for ABI. The mean value of these two measurements were separately recorded for each side. The maximum value of baPWV on the left and right sides was used. The minimum value of ABI on the left and right sides was used in the analysis.

### Other covariates

The demographic characteristics, smoking status, history of diseases, and medication uses were collected at baseline survey via a standard questionnaire by our trained staff. Smoking status was divided into current smoking or not. Anthropometric measurements were performed. Body mass index (BMI) was calculated as weight (in kilograms)/height squared (in meters squared). Overweight and obesity was defined as BMI ≥ 24.0 kg/m^2^ and ≥ 28.0 kg/m^2^ according to the BMI standard for Asian subjects [36]. Systolic blood pressure and diastolic blood pressure were presented as the average of two measurements on the right arm using a sphygmomanometer after resting for at least 10 min. Hypertension status was defined as systolic pressure ≥ 140 mmHg or diastolic pressure ≥ 90 mmHg, self-reported diagnosis history of hypertension, or use of antihypertensive medication [37]. Type 2 diabetes was defined as fasting glucose ≥ 7.0 mmol/L or using any glucose-lowering medication or self-reported diagnosis history of diabetes [38]. In this current study, there was no type 1 diabetes reported. Lipid-lowering medication referred to any use of statins or fibrates in this study.

### Statistical analysis

Statistical analysis was conducted from May to December 2021. The baseline characteristics of the study population by arteriosclerosis and atherosclerosis progression clusters were described, using medians (25th–75th percentiles) for continuous variables and frequencies (proportions) for categorical variables. The differences were compared by Kruskal-Wallis test or chi-squared test between three groups, as appropriate.

We estimated the joint progression trajectories of arteriosclerosis and atherosclerosis across the five biennial visits by baPWV and ABI, using group-based multi-trajectory modeling [39]. We fitted the joint changes of baPWV and ABI with age as the time-scale following previous studies [40, 41]. The group-based multi-trajectory model allows the identification of clusters of individuals following similar patterns through multiple visits using multi-variables. Varied models were considered to choose the optimal number of distinct groups and trajectory shape parameters (e.g., linear, quadratic, cubic) based on Bayesian information criteria (BIC) and Akaike information criterion (AIC). Sufficient sample sizes in each multi-trajectory group (> 5% of the sample) and clinical interpretation are important additional elements when determining the best model. Furthermore, we fitted the progression trajectory of baPWV and ABI separately using group-based trajectory modeling.

Unadjusted and adjusted ordinal logistics models were used to assess the associations of baseline lipid profiles (considered as both continuous and categorical variables) with arteriosclerosis and atherosclerosis progression clusters. Adjustments were made for age, sex, BMI, smoking status, systolic blood pressure, hypertension, diabetes, antihypertensive medication, and lipid-lowering treatment. Using the same models, we assessed the association of RC and LDL-C concordant/discordant groups with baPWV and ABI progression using the difference in percentile units, clinical cut-off points, and medians of RC and LDL-C. Finally, we performed three sensitivity analyses by excluding individuals on lipid-lowering therapy (*n* = 634), additionally adjusting for hsCRP (available in 2358 of 3186 individuals) and further adjusting for the change of systolic blood pressure during the follow-up period to explore the stability of our findings.

The group-based trajectory modeling technique was implemented using *Proc Traj* in Stata software version 14 (STATA Corp., TX, US). All other statistical analyses were performed with R software version 4.1.0 (R Foundation for Statistical Computing, Vienna, Austria). Two-sided *p* < 0.05 was considered statistically significant.

## Results

### Characteristics

The baseline characteristics of the participants are shown in Additional file 1: Table S1. Of 3186 individuals, the median (*P*_25_-*P*_75_) of age was 65.0 (57.0,75.0) years; 2445 (76.7%) were male; 829 (26.0%) with hypertension; and 337 (10.6%) with diabetes. Median levels were RC: 23.35 mg/dL, LDL-C: 114.86 mg/dL, non-HDL-C: 128.26 mg/dL; HDL-C: 48.52 mg/dL. The percentage of concordant RC and LDL-C was 23.5%, while 40.1% had discordantly low RC, and 36.4% had discordantly high RC. Proportions of concordance/discordance among individuals according to LDL-C clinical cut-off points are presented in Additional file 2: Fig. S1.

### Clusters of arteriosclerosis and atherosclerosis progression

**Fig. 1:**
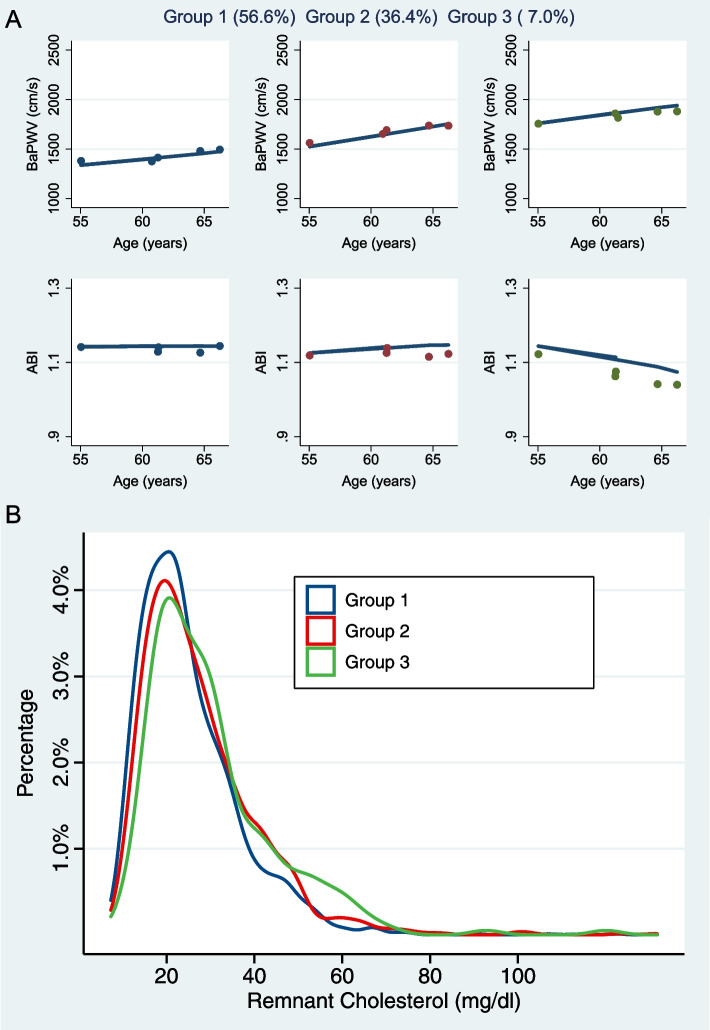
The distribution of remnant cholesterol among groups of arteriosclerosis and atherosclerosis progression clustered by multi-trajectory model. **A** Joint arteriosclerosis and atherosclerosis progression patterns clustered by multi-trajectory model. **B** Density distribution of remnant cholesterol in trajectory groups. Dots show group-specific mean observed levels, and solid lines represent fitted trajectories. baPWV, brachial-ankle pulse wave velocity; ABI, ankle-brachial index

Using the criteria mentioned above, we determined three clusters using the multi-trajectory model as stable baPWV/stable ABI (group 1: 56.6%), increasing baPWV/stable ABI (group 2: 36.4%), and increasing baPWV/decreasing ABI (group 3: 7.0%). The procedure of choosing the optimal group number and shape parameter for the final model was shown in Additional file 1: Table S2. Thus, the three groups represented a gradually increased risk of joint arteriosclerosis and atherosclerosis progression. Figure 1A shows the longitudinal joint trajectories of baPWV and ABI, and the percentages for each group. The characteristics of the three clusters are shown in Table 1. The RC (medians: 22.6 vs. 24.4 vs. 26.9 mg/dL, *p* < 0.001) and HDL-C (medians: 49.6 vs. 47.6 vs. 44.6 mg/dL, *p* < 0.001) were significantly distributed in three groups, whereas there were no significant differences for LDL-C (medians: 115.1 vs. 114.3 vs. 113.5 mg/dL, *p* = 0.852), or non-HDL-C (medians: 127.6 vs. 128.5 vs. 131.1 mg/dL, *p* = 0.208). The RC distribution according to the joint progression clusters is shown in Fig. 1B. We also derived a multi-group propensity score weighting model to infer the casual differences of lipid profiles. In the matched multi-trajectory clusters, RC concentrations were still significantly higher in group 2 and group 3, compared to group 1 (Additional file 1: Table S3). In addition, we fitted the progression trajectories of baPWV and ABI separately (Additional file 2: Fig. S2).

**Table 1:** Characteristics in multi-trajectory groups of joint baPWV and ABI

	Group 1^d^ (*n* = 1802)	Group 2 (*n* = 1160)	Group 3 (*n* = 224)
Age, years	64.00 [56.00, 73.00]	66.00 [58.00, 76.00]	66.00 [56.00, 77.00]
Female sex, *n* (%)	473 (26.2)	238 (20.5)	30 (13.4)
BMI, kg/m^2^	25.16 [23.29, 27.26]	25.48 [23.82, 27.25]	25.55 [23.87, 27.48]
BMI group, *n* (%)			
24.0 kg/m^2^	576 (33.4)	300 (27.1)	58 (27.4)
24.0–27.9 kg/m^2^	837 (48.5)	617 (55.7)	111 (52.4)
≥ 28.0 kg/m^2^	314 (18.2)	191 (17.2)	43 (20.3)
Current smoker, *n* (%)	432 (24.0)	280 (24.1)	45 (20.1)
SBP, mmHg	122.81 (15.91)	132.80 (16.47)	139.47 (17.04)
SBP change, mmHg	4.61 (23.55)	5.31 (22.97)	9.81 (25.62)
Hypertension, *n* (%) ^b^	413 (22.9)	345 (29.7)	71 (31.7)
Diabetes, *n* (%) ^c^	153 (8.5)	138 (11.9)	46 (20.5)
Antihypertensive medication, *n* (%)	386 (21.4)	328 (28.3)	64 (28.6)
Lipid-lowering medication use, *n* (%)	352 (19.5)	241 (20.8)	41 (18.3)
Triglycerides, mg/dL	113.09 [85.28, 152.94]	121.83 [91.90, 168.42]	134.56 [99.51, 179.19]
RC, mg/dL	22.60 [17.06, 30.51]	24.37 [18.38, 33.59]	26.91 [19.91, 35.19]
LDL-C, mg/dL	115.06 [97.42, 133.23]	114.26 [97.29, 135.44]	113.52 [94.60, 131.08]
HDL-C, mg/dL	49.64 [42.61, 58.69]	47.55 [40.98, 55.29]	44.58 [39.80, 51.24]
Non-HDL-C, mg/dL	127.64 [109.15, 146.36]	128.51 [108.82, 151.06]	131.11 [108.74, 149.44]
hsCRP, mg/L ^d^	0.76 [0.46, 1.42]	0.90 [0.52, 1.72]	1.01 [0.58, 1.92]

Continuous variables are reported as median (25th–75th percentile). Medians and proportions were compared using Kruskal–Wallis or chi-squared test

SI conversion factor: To convert RC, LDL-C, HDL-C, non-HDL-C to mmol/L, multiply by 0.02586; TG, multiply by 0.01129

*Abbreviations*: *CI* Confidence interval, *BMI* Body mass index, *SBP* Systolic blood pressure, *DBP* Diastolic blood pressure, *RC* Remnant cholesterol, *LDL-C* Low-density lipoprotein cholesterol, *HDL-C* High-density lipoprotein cholesterol, *hsCRP* High-sensitivity C-reactive protein

^a^Hypertension defined as SBP ≥ 140 mmHg or DBP ≥ 90 mmHg, self-reported diagnosis history of hypertension or use of antihypertensive medication

^b^Diabetes defined as fasting glucose ≥ 7.0 mmol/L or using any glucose-lowering medication or self-reported diagnosis history of diabetes

^c^hsCRP data only available in a part of overall population (2358 of 3186 subjects)

^d^Group 1 represents the stable baPWV/stable ABI, group 2 represents increasing baPWV/stable ABI, and group 3 represents increasing baPWV/decreasing ABI

### Association of lipid profiles with baPWV and ABI progression

In the current study, RC was weakly correlated with LDL-C (*β* = 0.061, *p* < 0.05) but moderately correlated with triglycerides (*β* = 0.792, *p* < 0.001) and HDL-C (*β* = − 0.461, *p* < 0.001) after adjusting for age and sex (Additional file 1: Table S4). The scatter plots between RC and LDL-C are presented in Additional file 2: Fig. S3.

**Fig. 2:**
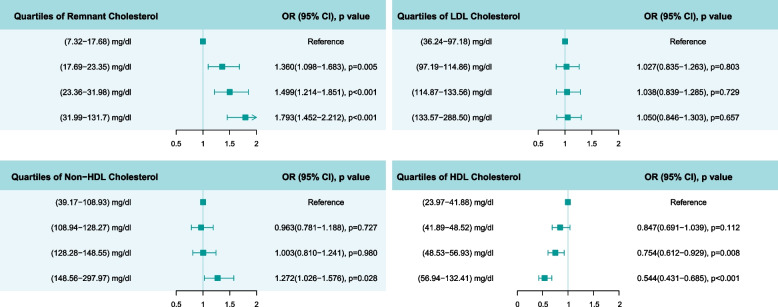
Association of cholesterol components with arteriosclerosis and atherosclerosis progression. Odds ratios (95% CI) were estimated by ordinal logistics regression models adjusted for age, sex, body mass index, smoking status, systolic pressure, hypertension, diabetes, antihypertensive medication, and lipid-lowering treatment

In the ordinal logistics models, we observed significant associations of triglycerides, RC, HDL-C, and non-HDL-C with joint baPWV and ABI progression (Table 2). The adjusted OR (95% CI) were 1.029 (1.019–1.039) for triglycerides per 10 mg/dL increase, 1.203 (1.132–1.278) for RC per 10 mg/dL increase, 0.905 (0.872–0.939) for HDL-C per 5 mg/dL increase, and 1.043 (1.016–1.070) for non-HDL-C per 10 mg/dL increase, respectively. The OR values of other covariates in the adjusted model are shown in Additional file 1: Table S5. We further examined the association of the quartile of lipid profiles with the joint baPWV and ABI progression trajectories (Fig. 2).

**Table 2:** Association of baseline lipid profiles with the increase of baPWV along with a decreasing ABI

	Unadjusted odds ratio (95% CI)	*p* value	Adjusted odds ratio (95% CI)	*p* value
Triglycerides, + 10 mg/dL	1.030 (1.021–1.039)	< 0.001	1.029 (1.019–1.039)	< 0.001
RC, + 10 mg/dL	1.207 (1.143–1.275)	< 0.001	1.203 (1.132–1.278)	< 0.001
LDL-C, + 10 mg/dL	1.007 (0.982–1.033)	0.585	1.025 (0.996–1.054)	0.091
HDL-C, + 5 mg/dL	0.894 (0.867–0.922)	< 0.001	0.905 (0.872–0.939)	< 0.001
Non-HDL-C, + 10 mg/dL	1.029 (1.005–1.053)	0.018	1.043 (1.016–1.070)	< 0.001

Odds ratio (OR) was estimated by ordinal logistics regression models adjusted for age, sex, body mass index, smoking status, systolic blood pressure, hypertension, diabetes, antihypertensive medication, and lipid-lowering treatment

*Abbreviations*: *CI* Confidence interval, *RC* Remnant cholesterol, *LDL-C* Low-density lipoprotein cholesterol, *HDL-C* High-density lipoprotein cholesterol

The associations of lipid profiles with separate baPWV and ABI progression trajectories are shown in Additional file 1: Table S6. We also observed significant associations of RC (per 10 mg/dL increase) with a higher level of baPWV and a lower level of ABI, and the adjusted OR were 1.154 (1.033–1.201) and 1.206 (1.114–1.301), respectively.

### Discordance analysis of RC and LDL-C

Compared to the concordant group, the discordantly low RC had a significant decreased risk of joint arteriosclerosis and atherosclerosis progression after adjusting the common CVD risk factors (adjusted OR: 0.733; 95% CI: 0.604–0.889). However, we did not observe a significantly increased risk in the discordantly high RC group (adjusted OR: 0.989; 95% CI: 0.816–1.199). At the cut-off points of 130 mg/dL for LDL-C and 24 mg/dL for RC, those with high RC (≥ 24 mg/dL) and low LDL-C (< 130 mg/dL) had an increased risk of arteriosclerosis and atherosclerosis progression (adjusted OR: 1.445; 95% CI: 1.210–1.725), compared to the group with both low RC (< 24 mg/dL) and LDL-C (< 130 mg/dL). Notably, individuals with low RC (< 24 mg/dL) and high LDL-C (≥ 130 mg/dL) had a borderline increased risk (adjusted OR: 1.276; 95% CI: 1.000–1.628). In addition, consistent results were observed when using the clinical cut-off points of 100 mg/dL for LDL-C and 17 mg/dL for RC (Table 3), and the median values for LDL-C (114.86 mg/dL) and RC (23.35 mg/dL) (Additional file 1: Table S7).

**Table 3:** Odds ratios (95% confidence interval) for the increasing baPWV along with decreasing ABI across LDL-C vs. remnant cholesterol concordant/discordant groups and according to clinical cut-off points

	Unadjusted odds ratio (95% CI)	Adjusted odds ratio (95% CI)
RC percentile minus LDL-C percentile		
Concordant (within 10 percentiles), *n* = 749	Reference	Reference
Discordantly low RC, *n* = 1277	0.701 (0.586–0.837)	0.733 (0.604–0.889)
Discordantly high RC, *n* = 1160	1.017 (0.85–1.217)	0.989 (0.816–1.199)
Cutpoints: LDL-C 130 mg/dL; RC 24 mg/dL		
LDL-C < 130 mg/dL and RC < 24 mg/dL, *n* = 1273	Reference	Reference
LDL-C ≥ 130 mg/dL and RC < 24 mg/dL, *n* = 393	1.229 (0.981–1.539)	1.276 (1.000–1.628)
LDL-C < 130 mg/dL and RC ≥ 24 mg/dL, *n* = 986	1.378 (1.169–1.626)	1.445 (1.210–1.725)
LDL-C ≥ 130 mg/dL and RC ≥ 24 mg/dL, *n* = 534	1.689 (1.385–2.06)	1.599 (1.289–1.983)
Cutpoints: LDL-C 100 mg/dL; RC 17 mg/dL		
LDL-C < 100 mg/dL and RC < 17 mg/dL, *n* = 301	Reference	Reference
LDL-C ≥ 100 mg/dL and RC < 17 mg/dL, *n* = 395	1.569 (1.154–2.132)	1.348 (0.966–1.879)
LDL-C < 100 mg/dL and RC ≥ 17 mg/dL, *n* = 610	1.686 (1.281–2.218)	1.604 (1.190–2.161)
LDL-C ≥ 100 mg/dL and RC ≥ 17 mg/dL, *n* = 1880	2.025 (1.601–2.561)	1.899 (1.464–2.463)

Odds ratio (OR) was estimated by ordinal logistics regression models adjusted for age, sex, body mass index, smoking status, systolic blood pressure, hypertension, diabetes, antihypertensive medication, lipid-lowering treatment

*Abbreviations*: *CI* Confidence interval, *RC* Remnant cholesterol, *LDL-C* Low-density lipoprotein cholesterol, *HDL-C* High-density lipoprotein cholesterol

(i) Concordant was defined as RC percentile and LDL-C percentile within ± 10 percentile units; (ii) discordantly low RC was defined as LDL-C percentile > RC percentile by 10 percentile units; and (iii) discordantly high RC was defined as RC percentile > LDL-C percentile by 10 percentile units

### Sensitivity analyses

We observed consistent results after excluding individuals with lipid-lowering medication use or additionally adjusting for hsCRP level and the change of systolic blood pressure during the follow-up period (Table 4). In addition, we performed subgroup analyses, and the associations of RC with joint arteriosclerosis and atherosclerosis progression remained consistent when stratified by age, sex, BMI level, hypertension, and diabetes status (Additional file 1: Table S8). We further calculated the alternative RC concentration using the Friedewald formula, and the association results were almost consistent (Additional file 1: Table S9).

**Table 4:** Associations of remnant cholesterol with the increasing baPWV along with decreasing ABI beyond LDL-C in sensitivity analyses

	Adjusted odds ratio (95% CI)		
	Sensitivity analysis I	Sensitivity analysis II	Sensitivity analysis III
RC concentration, + 10 mg/dL	1.220 (1.141–1.304)	1.176 (1.090–1.269)	1.256 (1.179–1.337)
Quartiles of RC			
Quartile 1	Reference	Reference	Reference
Quartile 2	1.251 (0.980–1.597)	1.311 (1.024–1.679)	1.398 (1.125–1.737)
Quartile 3	1.305 (1.017–1.675)	1.582 (1.239–2.020)	1.521 (1.228–1.884)
Quartile 4	1.862 (1.448–2.394)	1.951 (1.527–2.493)	1.789 (1.443–2.217)
RC percentile minus LDL-C percentile			
Concordant	Reference	Reference	Reference
Discordantly low RC	0.726 (0.585–0.901)	0.747 (0.598–0.934)	0.708 (0.583–0.862)
Discordantly high RC	1.047 (0.839–1.306)	0.979 (0.785–1.221)	0.970 (0.798–1.179)
Cut-off: LDL-C 130 mg/dL; RC 24 mg/dL			
LDL-C < 130 mg/dL and RC < 24 mg/dL	Reference	Reference	Reference
LDL-C ≥ 130 mg/dL and RC < 24 mg/dL	1.672 (1.085–2.578)	1.307 (0.896–1.907)	1.190 (0.928–1.525)
LDL-C < 130 mg/dL and RC ≥ 24 mg/dL	1.446 (1.170–1.787)	1.395 (1.111–1.753)	1.381 (1.154–1.652)
LDL-C ≥ 130 mg/dL and RC ≥ 24 mg/dL	1.559 (1.255–1.937)	1.385 (1.115–1.720)	1.606 (1.291–1.997)
Cut-off: LDL-C 100 mg/dL; RC 17 mg/dL			
LDL-C < 100 mg/dL and RC < 17 mg/dL	Reference	Reference	Reference
LDL-C ≥ 100 mg/dL and RC < 17 mg/dL	1.561 (1.07–2.277)	1.259 (0.876–1.810)	1.233 (0.879–1.728)
LDL-C < 100 mg/dL and RC ≥ 17 mg/dL	1.595 (1.154–2.205)	1.587 (1.129–2.231)	1.551 (1.146–2.100)
LDL-C ≥ 100 mg/dL and RC ≥ 17 mg/dL	2.026 (1.522–2.696)	1.784 (1.331–2.391)	1.940 (1.489–2.527)

Odds ratio (OR) was estimated by ordinal logistics regression models adjusted for age, sex, body mass index, smoking status, SBP, hypertension, diabetes, antihypertensive medication, lipid-lowering treatment

*Abbreviations*: *CI* Confidence interval, *RC* Remnant cholesterol, *LDL-C* Low-density lipoprotein cholesterol, *HDL-C* High-density lipoprotein cholesterol, *SBP* Systolic blood pressure

(i) Concordant was defined as RC percentile and LDL-C percentile within ± 10 percentile units; (ii) discordantly low RC was defined as LDL-C percentile > RC percentile by 10 percentile units; and (iii) discordantly high RC was defined as RC percentile > LDL-C percentile by 10 percentile units

Sensitivity analysis I: Individuals (*n* = 634) on lipid-lowering medication (referring to statins and fibrates) excluded from the analysis

Sensitivity analysis II: High-sensitivity C-reactive protein (hsCRP) was additionally adjusted

Sensitivity analysis III: Change of systolic blood pressure during the follow-up period was additionally adjusted

## Discussion

In this cohort study, we identified clusters representing distinct patterns of joint arteriosclerosis and atherosclerosis progression using a group-based multi-trajectory method (see Fig. 1). This method clusters distinct trajectory patterns considering more than one variable and is able to depict the joint progression of intra-correlated measurements. The baPWV measures the arterial stiffness level, and it is closely related with age and other CVD risk factors, while ABI as a noninvasive detection measure is widely used to diagnose peripheral artery disease in clinical practice [42]. We chose the optimal number (set as 3) of distinct groups and trajectory shape parameters (set as 2) based on BIC and AIC and identified three trajectory groups following distinct patterns of joint baPWV and ABI changes during a 10-years follow-up (all fitted *p* value < 0.001). Group 1 was characterized by stable baPWV and ABI, while group 2 showed a stable ABI but a steeper increase of baPWV, and group 3 had a strong increase of baPWV up to 2000 cm/s along with a decreasing ABI. Individuals with unfavorable joint changes of baPWV and ABI had higher blood pressure, BMI, triglycerides, hsCRP, and lower HDL-C and were more likely to suffer from diabetes. Combining the two indicators for trajectory analysis could identify high-risk groups of CVD and improve the early primary prevention of CVD.

We found that higher RC was significantly associated with the joint and separate arteriosclerosis and atherosclerosis progressions measured by baPWV and ABI after adjusting for traditional confounding factors and LDL-C. In addition, discordance analyses revealed that increased RC level differentiates individuals at a higher risk of arteriosclerosis and atherosclerosis progression, even in people with an optimal LDL-C level. Our findings are consistent with most previous studies. A longitudinal study reported that higher levels of RC, but not LDL-C, were associated with major adverse cardiovascular events independent of other risk factors among overweight or obese subjects at high cardiovascular risk [17]. Similar findings were observed that RC could predict atherosclerotic CVD beyond LDL-C [26]. Our study supplemented the evidence about RC and subclinical indicators of CVD risk in the general population. We found that RC, rather than LDL-C, was significantly associated with the 10-year joint progression of arteriosclerosis and atherosclerosis. Only two cross-sectional studies reported that RC was associated with higher baPWV alone [7, 29]. Our founding extended the data on the longitudinal association between RC and arterial stiffness. A Copenhagen study found that higher level of RC at baseline increased the risk of incident peripheral arterial disease [27]. Our study fitted multi-trajectories to describe the 10-year joint progression of arteriosclerosis and peripheral atherosclerosis. We revealed the relationship between RC and the joint progression of baPWV and ABI for the first time from the perspective of early primary prevention of CVD.

In real-world clinical practice, LDL-C is commonly considered as the primary therapy target both in the primary and secondary prevention of adverse CVD [43]. The fact is that after reducing LDL-C to recommended levels, there still exists a considerable residual risk of CVD and adverse outcomes [11]. This residual risk has been partly recognized as a result of the common lipid disorder characterized by high circulating triglycerides and low HDL-C with normal concentrations of LDL-C [44]. However, randomized controlled trials with HDL-C as a therapeutic target reported that the use of HDL-C modifying treatments had no significant effect on CVD [18]. A prospective cohort study among 15.8 million adults found that both low and high levels of HDL-C were associated with increased mortality from CVD, supporting that high HDL-C is not necessarily a sign of optimal cardiovascular health [45]. Thus, recent research focused on TRLs and the embedded cholesterol component [26]. Triglycerides can be easily metabolized in most cells [46]. Thus, it is hypothesized that the harmful component in TRLs is cholesterol rather than triglycerides [46], which was validated in our discordance analyses. Our results revealed that RC plays a key role in the pathological arteriosclerosis and atherosclerosis progression, even in patients with optimal LDL-C levels. In addition, RC yielded greater risk for arteriosclerosis and atherosclerosis progression than triglycerides. Previous study indicated that RC could increase the risk of CVD regardless of LDL-C level through discordance analyses [17, 26]. BaPWV and ABI are two validated predictive factors for CVD, which have potential to be more widely used as early markers of the primary prevention strategies for CVD [47]. Our conclusions extended these prior findings and confirmed the association of RC with joint arteriosclerosis and atherosclerosis progression. Interestingly, we found that the discordant low RC group was associated with decreased risk of arteriosclerosis and atherosclerosis, which may provide a time window for early CVD prevention, even for those with optimal LDL-C levels.

Although the exact mechanisms underlying the association of RC with arteriosclerosis and atherosclerosis still need to be established, several potential biological pathways can be proposed. Like low-density lipoprotein, particles containing RC in the blood flow accumulate through the endodermis and are absorbed by macrophages and smooth muscle cells, forming foam cells, which eventually become part of atherosclerotic plaques [48]. Due to the relatively large size of remnant lipoproteins compared with LDL, RC is more easily trapped and taken up by macrophages than LDL-C, leading to a faster formation of foam cells and arterial damage [49]. Our study also found that RC, not LDL-C, was significantly associated with the joint arteriosclerosis and atherosclerosis progression, indicating the atherogenic effect of RC. In addition, higher RC level was regarded as a risk factor of endothelial dysfunction, which may mediate the progression of arteriosclerosis and atherosclerosis [50]. Finally, genetic evidence indicated that elevated RC was associated with low-grade inflammation, thus promoting the progression of coronary burden, arterial stiffness, and atherosclerosis [20]. Of note, the results remained significant after additionally adjusting for hsCRP in our analysis (see Table 4), which indicated that the underlying mechanism remains to be further clarified.

There has been an increasing clinical interest in RC targeted interventions. Several studies showed that liraglutide, high-dose n-3 fatty acid supplementation, particularly icosapent ethyl and peroxisome proliferator-activated receptor alpha modulators could serve as novel candidates to reduce RC level [25, 51, 52]. However, the reported clinical benefits of RC-lowering therapy were distinct. A recent study suggested that using *icosapent ethyl* could reduce the concentrations of atherogenic remnant particle-cholesterol and concomitantly lessen the occurrence of certain cardiovascular events independent of statin treatment [51]. Another randomized controlled trial performed in patients with type 2 diabetes found that the incidence of cardiovascular events was not lower among those receiving pemafibrate, although pemafibrate could lower RC levels [53]. Notably, a randomized crossover study in patients of hyperlipidemia, atorvastatin, and simvastatin significantly reduced RC levels in addition to LDL-C. This may be another potential mechanism to explain the cardiovascular benefits from statins [54]. Future research should further explore more RC targeted interventions to slow down the progression of arteriosclerosis and atherosclerosis, thus reducing cardiovascular risk.

## Limitations

This cohort design with multiple surveys supplements the evidence about the longitudinal association of RC with joint arteriosclerosis and atherosclerosis progression using an innovative multi-trajectory modeling technique [39, 55]. However, the results should be interpreted in the context of limitations. First, RC concentration was not directly measured but obtained by calculation, which may deviate from the actual level. However, the calculated RC is closely correlated with the directly measured RC and is widely used in population studies [27, 56]. Second, the baPWV measures the stiffness of the elastic aorta and the muscular arteries, but only aortic stiffness is more closely related with CVD risk [57]. However, a meta-analysis showed that the measurement of baPWV could enhance the efficacy of predicting cardiovascular events, which was comparable with the Framingham risk score in 14,673 Japanese participants [58], indicating that baPWV level reflects the CVD risk, especially in the Asian population. In this current study, the cfPWV data were not available, and we were unable to compare the results between baPWV and cfPWV. Third, although we adjusted for some confounding factors, there is still a possibility of residual confounding bias. For example, the data of fish oil supplement were not collected in this study, which could have an effect on the RC level. Finally, the observed results require further validation in other populations.

## Conclusions

This longitudinal study indicated that RC is an early risk factor of the joint arteriosclerosis and atherosclerosis progressions independent of LDL-C in the general population, providing new evidence on the necessity of monitoring RC for promoting cardiovascular health. RC may serve as a potential prevention and intervention target of arteriosclerosis and atherosclerosis, even in people with an optimal LDL-C level.

## Supplementary Information

**Additional file 1: Table S1.** Baseline characteristics in male and female. **Table S2.** Diagnostic criteria for choosing the group number and shape parameter of the final multi-trajectory model. **Table S3.** Lipid component levels in the matched multi-trajectory groups. **Table S4.** Partial correlation matrix of lipid profiles and vascular measures adjusted for age and sex. **Table S5.** Full regression results of associations of remnant cholesterol and covariates with arteriosclerosis and atherosclerosis progression. **Table S6.** Associations of lipid profiles with separate baPWV and ABI trajectories. **Table S7.** Risk for arteriosclerosis and atherosclerosis progression according to the median and individualized cutpoints across LDL-C and remnant cholesterol. **Table S8.** Subgroup analysis of associations between remnant cholesterol level and arteriosclerosis and atherosclerosis progression in terms of age, sex, BMI, hypertension and diabetes. **Table S9.** Associations of alternative remnant cholesterol with arteriosclerosis and atherosclerosis progression beyond LDL-C.

**Additional file 2: Fig. S1.** Proportions of concordance/discordance among individuals according to LDL-C clinical cutpoints. **Fig. S2.** Progression trajectories of separate baPWV and ABI. **Fig. S3.** The scatter plots between remnant cholesterol and LDL cholesterol stratified by age and sex.

## Abbreviations

RC: Remnant cholesterol
CVD: Cardiovascular disease
BHMC: Beijing Health Management Cohort
baPWV: Brachial-ankle pulse wave velocity
ABI: Ankle brachial index
LDL-C: Low density lipoprotein cholesterol
TRLs: Triglyceride-rich lipoproteins
IDL: Intermediate density lipoprotein
VLDL: Very low density lipoprotein
hsCRP: High-sensitivity C-reactive protein
cIMT: Carotid intima-media thickness
