# Supplementary material for: Longitudinal association of remnant cholesterol with joint arteriosclerosis and atherosclerosis progression beyond LDL cholesterol
Source: BMC Med. 2023 Feb 6;21:42. doi: 10.1186/s12916-023-02733-w (PMC9903550; doi:10.1186/s12916-023-02733-w)
Supplement: Supplementary file 1 — Additional file 1: Table S1. Baseline characteristics in male and female. Table S2. Diagnostic criteria for choosing the group number and shape parameter of the final multi-trajectory model. Table S3. Lipid component levels in the matched multi-trajectory groups. Table S4. Partial correlation matrix of lipid profiles and vascular measures adjusted for age and sex. Table S5. Full regression results of associations of remnant cholesterol and covariates with arteriosclerosis and atherosclerosis progression. Table S6. Associations of lipid profiles with separate baPWV and ABI trajectories. Table S7. Risk for arteriosclerosis and atherosclerosis progression according to the median and individualized cutpoints across LDL-C and remnant cholesterol. Table S8. Subgroup analysis of associations between remnant cholesterol level and arteriosclerosis and atherosclerosis progression in terms of age, sex, BMI, hypertension and diabetes. Table S9. Associations of alternative remnant cholesterol with arteriosclerosis and atherosclerosis progression beyond LDL-C. [file 12916_2023_2733_MOESM1_ESM.docx]

**Additional file 1**

**Table S1**: Baseline characteristics in male and female.

|  | Overall (n=3186) | male (n=2445) | female (n=741) |
| --- | --- | --- | --- |
| Age, years | 65.00[57.00,75.00] | 65.00[58.00,76.00] | 64.00[56.00,71.00] |
| BMI, kg/m2 | 25.31[23.52,27.27] | 25.62[23.95,27.47] | 24.12[22.07,25.97] |
| BMI group |  |  |  |
| <24.0 kg/m2 | 934(30.7) | 597(25.5) | 337(47.5) |
| 24.0-27.9 kg/m2 | 1565(51.4) | 1282(54.9) | 283(39.9) |
| ≥28.0 kg/m2 | 548(18.0) | 458(19.6) | 90(12.7) |
| Current smoker, n (%) | 757(23.8) | 630(25.8) | 127(17.1) |
| Hypertension, n (%) | 829(26.0) | 668(27.3) | 161(21.7) |
| Diabetes, n (%) | 337(10.6) | 282(11.5) | 55(7.4) |
| Lipid-lowering  medication use, n (%) | 634(19.9) | 472(19.3) | 162(21.9) |
| Triglycerides, mg/dL | 116.77[88.38,160.37] | 120.20[90.90,165.50] | 106.99[81.74,145.53] |
| LDL-C, mg/dL | 114.86[97.18,133.56] | 113.18[95.97,132.51] | 119.17[103.80,137.01] |
| HDL-C, mg/dL | 48.52[41.88,56.93] | 46.55[40.50,53.45] | 57.22[48.97,65.82] |
| Non-HDL-C, mg/dL | 128.26[108.93,148.55] | 127.19[107.86,147.59] | 131.21[114.34,151.06] |
| Remnant-C, mg/dL | 23.35[17.68,31.98] | 24.04[18.18,32.90] | 21.37[16.35,29.06] |
| hsCRP, mg/L | 0.82[0.49,1.55] | 0.80[0.49,1.48] | 0.89[0.51,1.92] |

Continuous variables are reported as median (25th–75th percentile).

SI conversion factor: To convert RC, LDL-C, HDL-C, Non-HDL-C to mmol/L, multiply by 0.02586;TG, multiply by 0.01129.

Abbreviations: CI, confidence interval; BMI, body mass index; RC, remnant cholesterol; LDL-C, low-density lipoprotein cholesterol; HDL-C, high-density lipoprotein cholesterol; hsCRP: high-sensitivity C-reactive protein.

a hsCRP data only available in a part of overall population (2 358 of 3 186 subjects).

**Table S2**: Diagnostic criteria for choosing the group number and shape parameter of the final multi-trajectory model.

| Number of groups | BIC | AIC | Order | Trajectory group | Percentage (%) | P value for baPWV | P value for ABI |
| --- | --- | --- | --- | --- | --- | --- | --- |
| 3 | -70952.85 | -70886.11 | 2 | Group 1 | 56.56 | <0.0001 | <0.0001 |
|  |  |  | 2 | Group 2 | 36.41 | 0.0004 | <0.0001 |
|  |  |  | 2 | Group 3 | 7.03 | <0.0001 | <0.0001 |
| 3 | -70796.1 | -70711.17 | 3 | Group 1 | 59.92 | <0.0001 | 0.0165 |
|  |  |  | 3 | Group 2 | 12.27 | 0.1265 | 0.0036 |
|  |  |  | 3 | Group 3 | 27.81 | <0.0001 | 0.7732 |
| 4 | -70313.05 | -70225.09 | 2 | Group 1 | 37.58 | <0.0001 | <0.0001 |
|  |  |  | 2 | Group 2 | 40.57 | 0.3473 | <0.0001 |
|  |  |  | 2 | Group 3 | 9.88 | <0.0001 | <0.0001 |
|  |  |  | 2 | Group 4 | 11.97 | 0.1267 | <0.0001 |
| 4 | -70271.1 | -70158.87 | 3 | Group 1 | 34.06 | 0.0001 | 0.0132 |
|  |  |  | 3 | Group 2 | 41.77 | <0.0001 | 0.1312 |
|  |  |  | 3 | Group 3 | 10.39 | 0.2811 | 0.0174 |
|  |  |  | 3 | Group 4 | 13.77 | <0.0001 | 0.0514 |
| 5 | -70050.56 | -69941.36 | 2 | Group 1 | 18.87 | <0.0001 | <0.0001 |
|  |  |  | 2 | Group 2 | 27.01 | <0.0001 | <0.0001 |
|  |  |  | 2 | Group 3 | 38.78 | 0.2329 | <0.0001 |
|  |  |  | 2 | Group 4 | 4.33 | 0.8078 | <0.0001 |
|  |  |  | 2 | Group 5 | 11.02 | 0.0021 | <0.0001 |
| 5 | -70083.08 | -66943.55 | 3 | Group 1 | 30.32 | 0.9028 | 0.2272 |
|  |  |  | 3 | Group 2 | 30.68 | 0.0004 | 0.0661 |
|  |  |  | 3 | Group 3 | 20.87 | 0.3712 | 0.0007 |
|  |  |  | 3 | Group 4 | 2.60 | 0.4159 | 0.0001 |
|  |  |  | 3 | Group 5 | 15.53 | <0.0001 | 0.0708 |

Abbreviations: BIC, Bayesian Information Criterion; AIC, Akaike Information Criterion; baPWV, brachial-ankle pulse wave velocity; ABI, ankle brachial index.

**Table S3**: Lipid component levels in the matched multi-trajectory groups.

|  | Estimate | Standard Error | P value |
| --- | --- | --- | --- |
| RC, mg/dL |  |  |  |
| Group 2 (ref: Group 1) | 1.043 | 0.449 | 0.020 |
| Group 3 | 2.151 | 1.054 | 0.041 |
| Triglycerides, mg/dL |  |  |  |
| Group 2 (ref: Group 1) | 12.785 | 3.038 | 0.000 |
| Group 3 | 23.335 | 7.824 | 0.003 |
| LDL-C, mg/dL |  |  |  |
| Group 2 (ref: Group 1) | 1.263 | 1.073 | 0.239 |
| Group 3 | 2.517 | 2.824 | 0.373 |
| HDL-C, mg/dL |  |  |  |
| Group 2 (ref: Group 1) | -1.820 | 0.462 | 0.000 |
| Group 3 | -4.932 | 0.860 | 0.000 |
| Non-HDL-C, mg/dL |  |  |  |
| Group 2 (ref: Group 1) | 2.456 | 1.153 | 0.033 |
| Group 3 | 5.018 | 3.225 | 0.120 |

Abbreviations: RC, remnant cholesterol; LDL-C, low-density lipoprotein cholesterol; HDL-C, high-density lipoprotein cholesterol.

We performed multigroup propensity score weighting procedure using the tree-based regression model. The covariate set of age, sex, BMI, systolic pressure and pulse pressure were considered in this weighting procedure.

**Table S4**: Partial correlation matrix of lipid profiles and vascular measures adjusted for age and sex.

|  | RC | LDL-C | TG | HDL-C |
| --- | --- | --- | --- | --- |
| RC | - | 0.061* | 0.792*** | -0.461*** |
| LDL-C | 0.061* | - | 0.053** | 0.051** |
| TG | 0.792*** | 0.053** | - | -0.41*** |
| HDL-C | -0.461*** | 0.051** | -0.41*** | - |

Abbreviations: RC, remnant cholesterol; LDL-C, low-density lipoprotein cholesterol; TG, triglycerides; HDL-C, high-density lipoprotein cholesterol.

**Table S5**: Full regression results of associations of remnant cholesterol and covariates with arteriosclerosis and atherosclerosis progression.

|  | Adjusted Odds Ratio  (95% CI) | p value |
| --- | --- | --- |
| RC as continuous variable: +10 mg/dl | 1.203(1.132-1.278) | <0.001 |
| age, years | 0.99(0.983-0.997) | 0.007 |
| male (ref: female) | 1.143(0.951-1.375) | 0.154 |
| BMI, kg/m^2^ | 0.968(0.943-0.994) | 0.015 |
| Current smoker | 0.902(0.757-1.076) | 0.252 |
| Hypertension | 0.878(0.491-1.57) | 0.662 |
| Diabetes | 1.813(1.406-2.337) | <0.001 |
| Lipid-lowering medication use | 0.806(0.647-1.004) | 0.054 |
| Antihypertensive medication | 1.254(0.692-2.275) | 0.456 |
| Systolic blood pressure | 1.044(1.039-1.049) | <0.001 |
| RC as categorical variable: Quartile group |  |  |
| (7.32-17.68) mg/dL | Reference |  |
| (17.69-23.35) mg/dL | 1.360(1.098-1.683) | 0.005 |
| (23.36-31.98) mg/dL | 1.499(1.214-1.851) | <0.001 |
| (31.99-131.7) mg/dL | 1.793(1.452-2.212) | <0.001 |
| age, years | 0.985(0.978-0.992) | <0.001 |
| male (ref: female) | 1.167(0.970-1.403) | 0.101 |
| BMI, kg/m^2^ | 0.982(0.957-1.007) | 0.164 |
| Current smoker | 0.920(0.772-1.096) | 0.350 |
| Hypertension | 0.878(0.492-1.564) | 0.658 |
| Diabetes | 1.794(1.391-2.313) | <0.001 |
| Lipid-lowering medication use | 0.784(0.629-0.978) | 0.031 |
| Antihypertensive medication | 1.258(0.695-2.276) | 0.448 |
| Systolic blood pressure | 1.045(1.040-1.050) | <0.001 |

Odds ratio (OR) was estimated by ordinal logistics regression models.

Abbreviations: CI, confidence interval; RC, remnant cholesterol; BMI, body mass index.

Arteriosclerosis and atherosclerosis progression refers to the increase of baPWV along with a decreasing ABI.

**Table S6:** Associations of lipid profiles with separate baPWV and ABI trajectories.

|  | baPWV trajectory | | ABI trajectory | |
| --- | --- | --- | --- | --- |
|  | Adjusted Odds Ratio  (95% CI) | P value | Adjusted Odds Ratio  (95% CI) | P value |
| Triglycerides, +10 mg/dL | 1.023(1.012-1.031) | <0.001 | 1.039(1.019-1.050) | <0.001 |
| RC, +10 mg/dL | 1.154(1.033-1.201) | <0.001 | 1.206(1.114-1.301) | <0.001 |
| LDL-C, +10 mg/dL | 1.001(0.978-1.029) | 0.518 | 1.036(0.917-1.093) | 0.112 |
| HDL-C, +5 mg/dL | 0.84(0.787-0.862) | <0.001 | 0.901(0.869-0.932) | <0.001 |
| Non-HDL-C, +10 mg/dL | 1.038(1.012-1.061) | 0.028 | 1.067(1.042-1.095) | <0.001 |

Odds ratio (OR) was estimated by ordinal logistics regression models adjusted for age, sex, body mass index, smoking status, systolic blood pressure, hypertension, diabetes, antihypertensive medication, lipid-lowering treatment.

Abbreviations: CI, confidence interval; RC, remnant cholesterol; LDL-C, low-density lipoprotein cholesterol; HDL-C, high-density lipoprotein cholesterol.

**Table S7**: Risk for arteriosclerosis and atherosclerosis progression according to the median and individualized cutpoints across LDL-C and remnant cholesterol.

|  | Adjusted Odds Ratio  (95% CI) |
| --- | --- |
| Cutpoints: median value of LDL-C and RC |  |
| LDL-C <114.86 mg/dL & RC <23.35 mg/dL | Reference |
| LDL-C ≥114.86 mg/dL & RC <23.35 mg/dL | 1.104(0.893-2.444) |
| LDL-C <114.86 mg/dL & RC ≥23.35 mg/dL | 1.603(1.306-3.690) |
| LDL-C ≥114.86 mg/dL & RC ≥23.35 mg/dL | 1.808(1.486-4.420) |

Odds ratio (OR) was estimated by ordinal logistics regression models adjusted for age, sex, body mass index, smoking status, systolic blood pressure, hypertension, diabetes, antihypertensive medication, lipid-lowering treatment.

Abbreviations: CI, confidence interval; RC, remnant cholesterol; LDL-C, low-density lipoprotein cholesterol.

Arteriosclerosis and atherosclerosis progression refers to the increase of baPWV along with a decreasing ABI.

**Table S8**: Subgroup analysis of associations between remnant cholesterol level and arteriosclerosis and atherosclerosis progression in terms of age, sex, BMI, hypertension and diabetes.

|  | Adjusted Odds Ratio (95% CI) |
| --- | --- |
| RC concentration, +10 mg/dL |  |
| Age |  |
| < 60 years | 1.304(1.183-1.438) |
| ≥ 60 years | 1.195(1.107-1.290) |
| Sex |  |
| male | 1.231(1.152-1.316) |
| female | 1.296(1.130-1.486) |
| BMI level |  |
| <24.0 km/m^2^ | 1.401(1.230-1.596) |
| ≥ 24.0 km/m^2^ | 1.196(1.119-1.278) |
| Hypertension ^a^ |  |
| yes | 1.126(1.008-1.257) |
| no | 1.282(1.194-1.377) |
| Diabetes ^b^ |  |
| yes | 1.154(1.104-1.315) |
| no | 1.243(1.201-1.309) |

Odds ratio (OR) was estimated by ordinal logistics regression models adjusted for age, sex, body mass index, smoking status, systolic blood pressure, hypertension, diabetes, antihypertensive medication, lipid-lowering treatment.

Abbreviations: CI, confidence interval; RC, remnant cholesterol; BMI, body mass index.

^a^ Hypertension defined as SBP ≥140 mmHg or DBP ≥90 mmHg, self-reported diagnosis history of hypertension or use of antihypertensive medication.

^b^ diabetes defined as fasting glucose ≥7.0 mmol/L or using any glucose-lowering medication or self-reported diagnosis history of diabetes.

Arteriosclerosis and atherosclerosis progression refers to the increase of baPWV along with a decreasing ABI.

**Table S9**: Associations of alternative remnant cholesterol with arteriosclerosis and atherosclerosis progression beyond LDL-C.

|  | Unadjusted Odds Ratio (95% CI) | p value | Adjusted Odds Ratio (95% CI) | p value |
| --- | --- | --- | --- | --- |
| RC concentration ^a^, +10 mg/dL | 1.139(1.073-1.210) | <0.001 | 1.156(1.083-1.234) | <0.001 |
| Quartiles of RC |  |  |  |  |
| (2.00-7.64) mg/dL | Reference |  |  |  |
| (7.65-11.89) mg/dL | 1.401(1.147-1.710) | 0.001 | 1.536(1.237-1.906) | <0.001 |
| (11.90-17.49) mg/dL | 1.596(1.308-1.948) | <0.001 | 1.637(1.319-2.031) | <0.001 |
| (17.50-131.7) mg/dL | 1.731(1.420-2.111) | <0.001 | 1.749(1.411-2.169) | <0.001 |
| RC percentile minus LDL-C percentile |  |  |  |  |
| Concordant | Reference |  |  |  |
| Discordantly low RC | 0.791(0.656-0.954) | 0.014 | 0.762(0.622-0.935) | 0.009 |
| Discordantly high RC | 0.887(0.736-1.070) | 0.210 | 0.855(0.699-1.047) | 0.130 |
| Cutpoints: LDL-C 130 mg/dL; RC 24 mg/dL |  |  |  |  |
| LDL-C <130 mg/dL & RC <24 mg/dL | Reference |  |  |  |
| LDL-C ≥130 mg/dL & RC <24 mg/dL | 1.179(0.992-1.401) | 0.061 | 1.150(0.952-1.389) | 0.148 |
| LDL-C <130 mg/dL & RC ≥24 mg/dL | 1.480(1.013-2.162) | 0.043 | 1.506(0.992-2.286) | 0.055 |
| LDL-C ≥130 mg/dL & RC ≥24 mg/dL | 1.493(1.164-1.915) | 0.002 | 1.562(1.189-2.052) | 0.001 |
| Cutpoints: LDL-C 100 mg/dL; RC 17 mg/dL |  |  |  |  |
| LDL-C <100 mg/dL & RC <17 mg/dL | Reference |  |  |  |
| LDL-C ≥100 mg/dL & RC <17 mg/dL | 1.515(0.871-2.637) | 0.142 | 1.788(0.999-3.201) | 0.051 |
| LDL-C <100 mg/dL & RC ≥17 mg/dL | 1.269(1.070-1.506) | 0.006 | 1.294(1.070-1.564) | 0.008 |
| LDL-C ≥100 mg/dL & RC ≥17 mg/dL | 1.608(1.326-1.950) | <0.001 | 1.719(1.385-2.134) | <0.001 |

Odds ratio (OR) was estimated by ordinal logistics regression models adjusted for age, sex, body mass index, smoking status, systolic blood pressure, hypertension, diabetes, antihypertensive medication, lipid-lowering treatment.

Abbreviations: CI, confidence interval; RC, remnant cholesterol; LDL-C, low-density lipoprotein cholesterol; HDL-C, high-density lipoprotein cholesterol.

1. Concordant was defined as RC percentile and LDL-C percentile within ±10 percentile units; (ii) Discordantly low RC was defined as LDL-C percentile > RC percentile by 10 percentile units; and (iii) Discordantly high RC was defined as RC percentile > LDL-C percentile by 10 percentile units.

^a^ RC concentration was determined as total cholesterol concentration minus HDL-C minus the calculated LDL-C, which is derived by Friedewald formula at triglyceride < 400 mg/dL in the sensitivity analyses.

Arteriosclerosis and atherosclerosis progression refers to the increase of baPWV along with a decreasing ABI.
