# Supplementary material for: Longitudinal association of remnant cholesterol with joint arteriosclerosis and atherosclerosis progression beyond LDL cholesterol
Source: BMC Med. 2023 Feb 6;21:42. doi: 10.1186/s12916-023-02733-w (PMC9903550; doi:10.1186/s12916-023-02733-w)
Supplement: Supplementary file 2 — Additional file 2: Fig. S1. Proportions of concordance/discordance among individuals according to LDL-C clinical cutpoints. Fig. S2. Progression trajectories of separate baPWV and ABI. Fig. S3. The scatter plots between remnant cholesterol and LDL cholesterol stratified by age and sex. [file 12916_2023_2733_MOESM2_ESM.docx]

**Additional file 2**


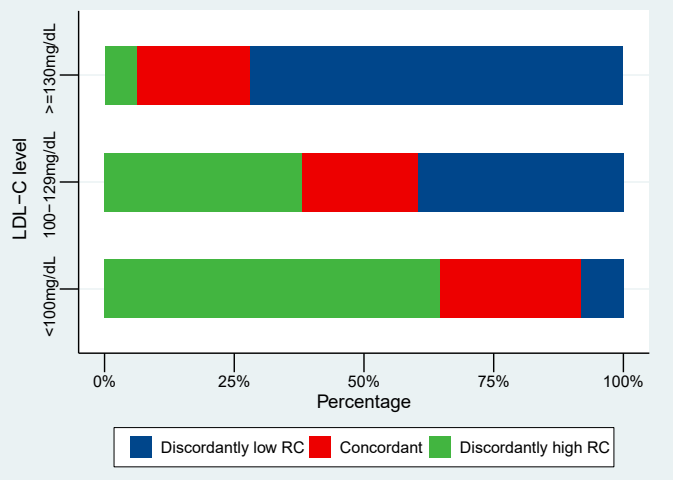


**Figure S1**: Proportions of concordance/discordance among individuals according to LDL-C clinical cutpoints.

Abbreviations: RC, remnant cholesterol; LDL-C, low-density lipoprotein cholesterol.

1. Concordant was defined as RC percentile and LDL-C percentile within ±10 percentile units; (ii) Discordantly low RC was defined as LDL-C percentile > RC percentile by 10 percentile units; and (iii) Discordantly high RC was defined as RC percentile > LDL-C percentile by 10 percentile units.

**
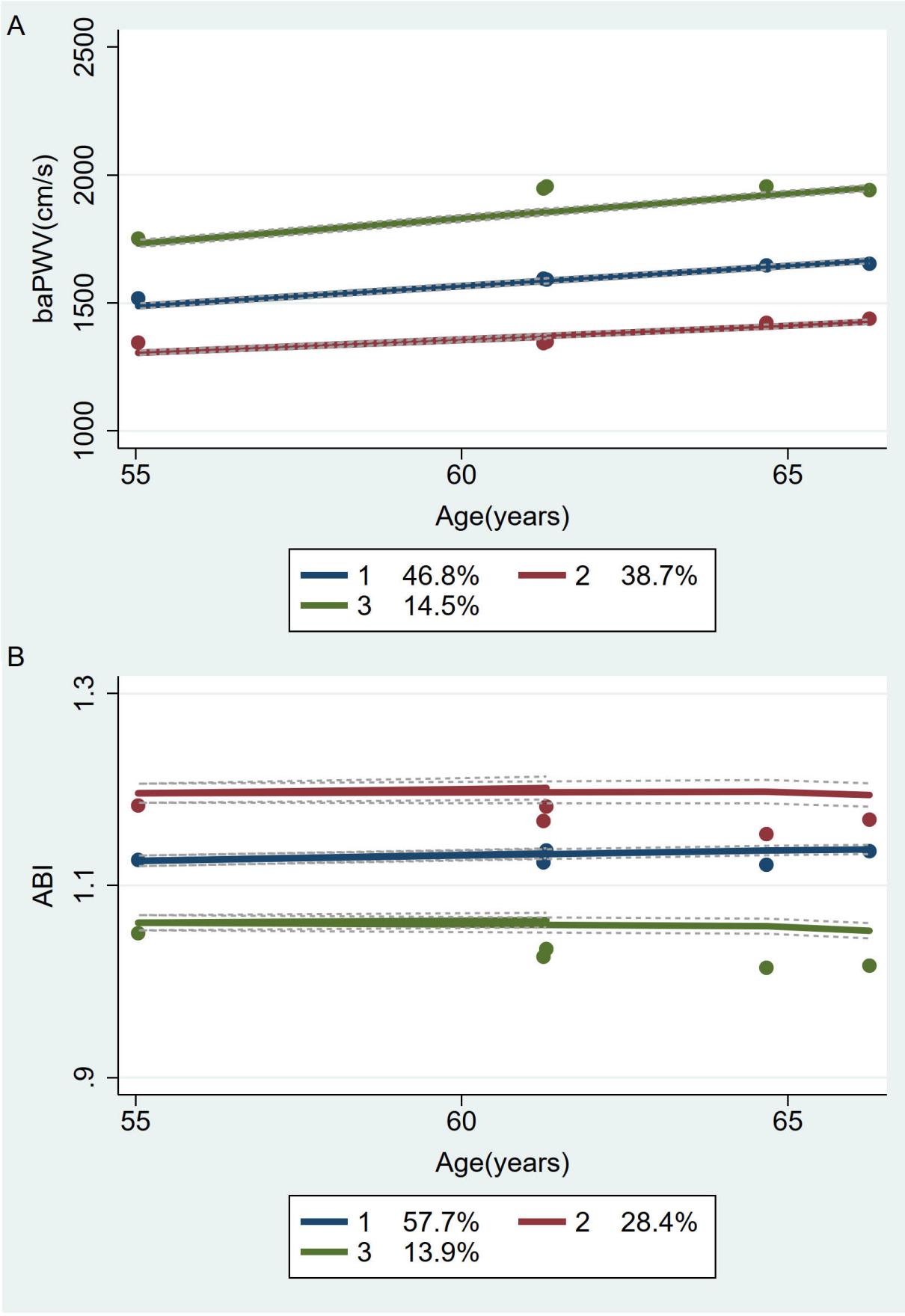
**

**Figure S2**: Progression trajectories of separate baPWV and ABI.


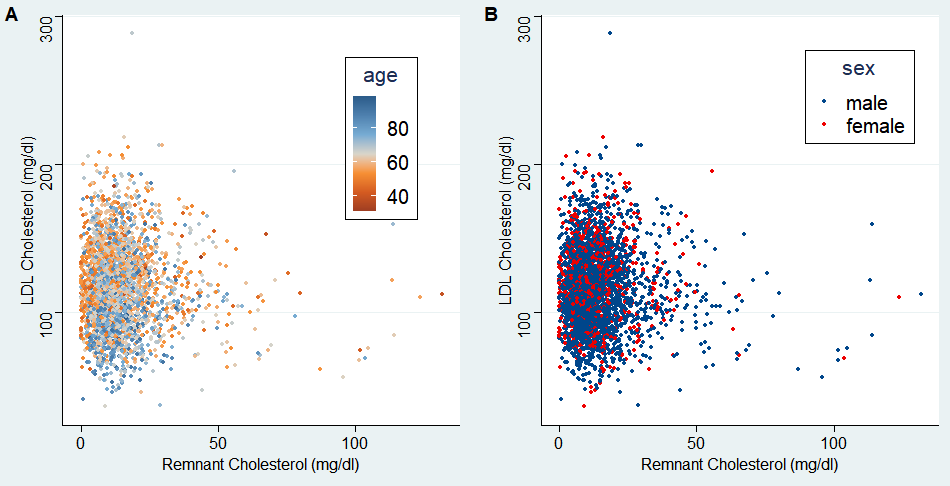


**Figure S3**: The scatter plots between remnant cholesterol and LDL cholesterol stratified by age and sex.
